# Coexistence of type 1 and type 2 diabetes mellitus: a case report of “double” diabetes in a 17-year-old Nigerian girl

**DOI:** 10.11604/pamj.2020.37.35.25191

**Published:** 2020-09-08

**Authors:** Michael Adeyemi Olamoyegun, Oluwabukola Ayodele Ala, Ejiofor Ugwu

**Affiliations:** 1 Department of Medicine, Endocrinology, Diabetes and Metabolism Unit, Ladoke Akintola University of Technology, Ladoke Akintola University of Technology Teaching Hospital, Ogbomoso, Oyo State, Nigeria,; 2 Department of Medicine, Bowen University, Bowen University Teaching Hospital, Ogbomoso, Oyo State, Nigeria,; 3 Department of Medicine, Enugu State University of Technology, Enugu, Enugu State, Nigeria

**Keywords:** Type 1 diabetes, type 2 diabetes, double diabetes, hybrid diabetes, adolescent, Nigeria

## Abstract

Double diabetes otherwise known as hybrid diabetes, a new variant, is a combination of both type 1 and type 2 diabetes in children and adolescents. It is a diabetes variant increasing in prevalence in developed countries because of epidemic obesity among children and adolescents but extremely rare in developing countries. Double diabetes is characterized by features of both type 1 (diabetes auto-antibodies) and type 2 (obesity and insulin resistance). This occurrence can either develop on a background of type 1 diabetes due to an abnormal increase in weight from physiological growth spurt in adolescents or from high insulin dosage developing on a background of type 2 diabetes. The variant has been linked to possible increased cardiovascular risks and worsened morbidity including poor glycaemic control. Here, we report a case of a 17-year-old girl who developed features of type 2 diabetes on a background of type 1 diagnosed 6 years after T1D diagnosis.

## Introduction

Diabetes mellitus is a metabolic disorder characterized by chronic hyperglycaemia (fasting plasma glucose ≥126 mg/dl or random plasma glucose ≥200mg/dl or HbA1c ≥6.5%) due to defects in insulin secretion and/or insulin action [1]. The major classification of diabetes mellitus are type 1 (T1D), type 2 (T2D), other specific types and gestational diabetes mellitus (GDM). Classically, a patient with a positive history of diabetes in first degree relatives, obesity/overweight, acanthosis nigrican, absent of both diabetes-associated auto-antibodies (DAA) and ketone bodies, with symptoms of polyuria, polydipsia, and polyphagia is diagnosed with T2D [2]. Conversely, T1D patients mostly have thin habitus, ketonaemia and diabetes associated autoantibodies [3]. Also, previously the distinction between type 1 and type 2 diabetes has been based on the age at onset of the disease, the relative or absolute insulin deficiency and dependence on insulin or not since diabetes diagnosis [4,5]. However, with the rising incidence of obesity among children and adolescents, T1D and T2D cannot be easily differentiated on the basis of only clinical features and causal factors, as beta-cell inefficiency is important and central in the pathogenesis of both conditions.

There is evidence that supports the hypothesis that T1D (fast diabetes), similarly to T2D (slow diabetes) will eventually cause insulin resistance, with obesity contributing significantly in its development [6]. A different type of diabetes has been reported in children and adolescent, designated as double diabetes, or hybrid diabetes. Double diabetes (DD) represents an entity presenting in childhood and adolescence, which combines characteristics of T1D (positive DAA) and T2D (obesity and insulin resistance), with a strong family history of T1D and T2D, who progress rapidly to insulin dependence or occasionally to reduction in insulin dosage with addition of insulin-sensitizer medications especially metformin [7]. An adolescent with pre-existing T1D or T2D can have features of DD which could be simultaneous with the initial diabetes diagnosis or develop consecutively over a period of time [3]. For example, in a patient with pre-existing T1D, the development of features in keeping with T2D is greatly influenced by the rapidity of weight gain/obesity and genetic factors. A typical patient is usually overweight or obese and requires high insulin doses to achieve/maintain euglycaemia as a result of insulin resistance associated with obesity [7,8].

Some patients may have additional metabolic features including metabolic syndrome (poor glycaemic control, hypertension, dyslipidaemia). Female adolescent patients may present with polycystic ovarian syndrome (PCOS). The prevalence of double diabetes is unknown [9], however, studies from the developed countries revealed that about 25% of children with T1D are either overweight or obese, a feature that is uncommon among children with type 1 diabetes in Nigeria and most developing countries [10]. On the contrary, about 35% of children and adolescents with T2D have at least one diabetes-associated antibody [11]. Here, we report a teenage girl who presented initially with features of T1D characterized by positive DAA, but within 6 years of diagnosis developed features consistent with insulin resistance like obesity and acanthosis nigricans, a feature suggestive of “double” diabetes (DD) a new variant with overlapping of type 1 and type 2 diabetes. There is paucity of data on the occurrence of double diabetes in Africa especially in Nigeria. An internet search on the reported case of double diabetes did not show any result. This is therefore, to the best of our knowledge the first reported diagnosis of double diabetes in Nigeria.

## Patient and observation

**Figure 1:**
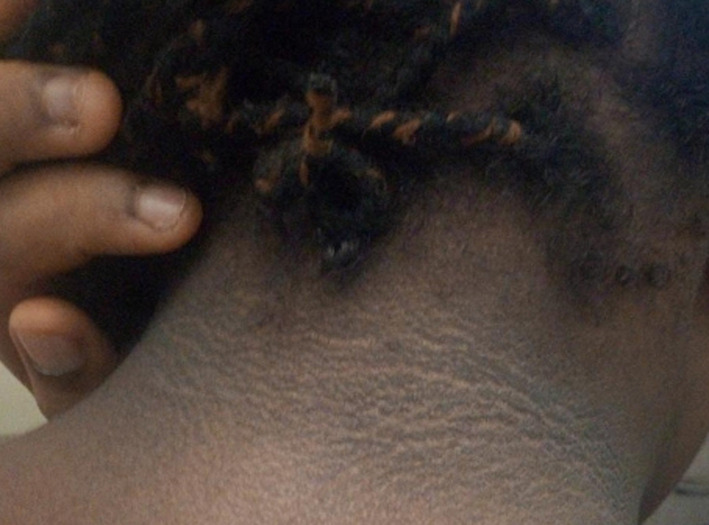
acanthosis nigricans around the neck of the patient

A diagnosis of type 1 diabetes was made in a 17-year-old girl after she presented acutely with features consistent with diabetic keto-acidosis at the age of 11 years. She had presented with osmotic symptoms, a random blood glucose level of 448mg/dl (28.4mmol/L), ketonuria (3+) and glycated hemoglobin (HbA1C-11.0%). The diagnosis of type 1 diabetes was made based on the high titre levels of auto-antibodies (anti-glutamic acid decarboxylase and anti-insulinoma associated antibodies) and low level of serum C-peptide, (Table 1). The patient´s body mass index (BMI) at the time of diagnosis was 23.8kg/m^2^with no markers suggestive of insulin resistance like acanthosis nigricans. Screening for antibodies associated with possible autoimmune thyroiditis was negative. Also there was no evidence of dyslipidaemia and blood pressure was within normal range. She was put on insulin treatment, (total daily insulin of 92-122units/day) with subcutaneous insulin glargine (a long-acting insulin analogue) and regular insulin at bed time and before three main meals respectively since discharged from hospital at diagnosis. She was the second of four siblings of her non-diabetic parents but had siblings who had type 1 diabetes. There was no history or clinical evidence suggestive of PCOS as her menstrual cycle was regular with no hirsutism or acne. However, 6 years post-diagnosis, she was noticed to have developed diffuse widespread acanthosis nigricans around the neck and axillary region indicating insulin resistance (Figure 1) and her weight increased significantly compared to when she was diagnosed of type 1 diabetes 6 years earlier. Her present BMI was 33.4 kg/m^2^, however her BP, fasting lipids profile were all normal. Hence, with these new features in the patient, she was diagnosed with type 2 diabetes on a background of type 1 diabetes (“double diabetes”). Moreover, she was counselled on lifestyle modification (diet and exercise) and metformin 1g twice daily added to her insulin. Within a period of 2 months, her daily insulin requirement reduced from 92-122 units to 48 units with significant improvement in her glycaemic profiles, her latest HbA1C was 8.3%.

**Table 1:** comparison between clinical and laboratory characteristics at initial presentation and 6 years post-diagnosis

Characteristics	At initial presentation (at diagnosis)	6 years post-diagnosis
Age (years)	11	17
DKA	Present	Absent
BMI (kg/m^2^)	23	33.4
Fasting C-peptide (ng/ml) [0.78-1.89]	0.17	0.09
Anti-GAD (U/ml) [0.0-10.0]	1208.8	1158
Anti-IA2 (U/ml)	Increased	High
Anti-thyroid Abs	Negative	Negative
HbA1C	11.0%	8.3%
Treatment	Insulin Sc	Life> Metformin + Insulin
Total daily insulin dose(Units)	92-122	48

DKA- diabetic ketoacidosis; BMI-body mass index; HbA1C-glycated hemoglobin; GAD-glutamic acid decarboxylase; IA2-insulinoma associated antibodies; Abs-antibodies

## Discussion

T1D is one of the most common chronic metabolic diseases diagnosed in children accounting for 80-90% of cases of diabetes in them [12]. The actual incidence and prevalence of T1D are still unknown in Nigeria. However, its incidence is said to be increasing in developed countries and this increase is said to be due partly to the high incidence of excessive weight gain in the young age group. Globally, epidemiological studies have shown the incidence of T1D rising annually by 2-5% [13] with overweight or obese children developing T1D at younger ages than normal weight children [14]. The diagnosis of T1D is made in the presence of auto antibodies against islet cells, insulin (IAA) glutamic acid decarboxylase (GAD/GAD65), protein tyrosine phosphatase-related islet antigen 2 (IA2/IA2β) and zinc transporter protein (ZnT8A) in the patient´s blood [14]. On the other hand, although type 2 diabetes is mostly found in adults, however there has been a rising trend in the incidence of young-onset T2D in adolescents and children [15]. Of particular interest is the recent recognition of a new type of diabetes in adolescents and children (double diabetes), characterized by a pooling of markers of both T1D and T2D. Our patient was a female teenager who initially presented with features consistent with T1D including DKA, presence of auto antibodies to GAD and IA-2 with almost undetectable level of serum C-peptide. However, she later developed obesity, insulin resistance and significantly responded to oral insulin sensitizer-metformin all of which favored the diagnosis of T2D.

The combined presence of anti-GAD antibodies, anti-insulin antibodies and features of type 2 diabetes eventually qualified her for the diagnosis of double diabetes. It has been shown that DD can be a significant occurrence in adolescents/young adults with diabetes (11-19 years old) due to rapid weight gain and insulin resistance resulting from insulin treatmen [16]. The use of exogenous insulin in the management of T1D fail to perfectly simulate the physiologic endogenous insulin secretion thereby exposing patients with type 1 diabetes to a state of persistent hyperinsulinaemia. This invariably can lead to weight gain and features in keeping with insulin resistance. Insulin dose, in this case, could be used as a surrogate marker of this hyperinsulinaemic environment, comparable to plasma insulin in individuals without diabetes but with metabolic syndrome. Interestingly, this was the scenario in our patient who developed progressive weight gain, acanthosis nigricans and required increasing dose of insulin, all in keeping with IR. It is the development of this T2D phenotype superimposed on T1D which had been diagnosed six years earlier that earned her the diagnosis of DD at the age of 17 years. Weight gain is known to be a common occurrence in adolescents with T1D after reaching adult age and this might further worsen insulin resistance. This progressive accumulation of weight is probably due to high dose of insulin which the patients are exposed to in an attempt to achieve good glycaemic control. Hence, type 1 diabetes individuals who are on high dose of insulin should be monitored closely for possible development of type 2 diabetes features and hence double diabetes especially if individual with type 1 diabetes is overweight or even obese.

This reported case is similar to a previous case of a 13-year-old girl with T1D, but with obesity leading to insulin resistance. Patient was managed with a combination of metformin and very high insulin doses [17]. Therefore, environmental factors especially significant weight gain could possibly result in the development of hybrid diabetes by their effect on the disease processes of T1D and T2D. A differential diagnosis considered in this patient was latent autoimmune diabetes mellitus (LADA), a clinical entity which consists of a heterogeneous group of patients sharing clinical, metabolic and genetic characteristics of both types 1 and type 2 diabetes. These patients have a slow onset type of diabetes, having initially the clinical and laboratory patterns of T2D in addition to the presence of DAA, but progress to insulin dependence more rapidly than in T2D, after a period of time ranging from months to few years after diagnosis [18]. LADA are of 2 types; type 1 which has high titer of auto antibodies along with low C-peptide level and type 2 with normal C-peptide level and insulin resistance [19]. Unlike LADA with slow progression, our patient presented acutely with ketoacidosis at the time of diagnosis of T1D. Over time, she maintained low C-peptide level and developed features of insulin resistance as manifested by acanthosis nigricans thus excluding both types of latent autoimmune diabetes mellitus. The present classification of LADA includes only adult patients >30 years old [20].

However, there is two previous reports [21,22] of three 8-9 year old children with latent autoimmune diabetes in children (LADC) because of the phenotypic similarity to LADA. Our patient had all the aforementioned characteristic features of LADA type 1, as she had two positive DAA and significantly reduced C-peptide levels. However, LADA, type 1 is not marked by the presence of overweight/obesity and insulin resistance [23], which was a persistent characteristic of our patient. Our patient responded well to lifestyle modifications and metformin 1gm twice daily with eventual significant reduction in her insulin dosage. The Food and Drug Administration (FDA) has approved the use of metformin in children with T2D in recent times, also used as adjunct to insulin therapy in adolescent with T1D having poor glycaemic control and insulin resistance (double diabetes) [24] as noted in our patient. With the rising incidence and prevalence of obesity worldwide, we envisage that many children with T1D will eventually also become insulin resistant. It has been shown that the coexistence of both T1D and T2D in an individual almost always increase the risk of diabetes complications in such individuals with diagnosis of double diabetes [25]. Therefore, these individuals are potentially at higher risk for both the acute diabetes complications (metabolic) and chronic diabetes complications (microvascular and macrovascular complications) of both types of diabetes [25]. This “double-hit” effect will further worsen health outcomes and put undue pressure on the national healthcare budget, which is barely existing in the many developing countries. Thus, an appropriate management protocol is required at this stage to improve glycaemic control in this “special” burgeoning sub-population.

## Conclusion

A new expression of diabetes mellitus, classified as double diabetes, resulting from the global pandemic of obesity in children and adolescents encompassed autoimmune entity T1D and the metabolic component of T2D. Presently, there is no consensus on the best therapeutic approach for this new variant of diabetes mellitus although use of weight-losing anti-diabetic agents could be a good option in addition to insulin therapy.
